# Proposal and Initial Validation of a Brief Questionnaire for Epidemiological Assessment of Low Back Pain

**DOI:** 10.1155/prm/6203306

**Published:** 2026-08-25

**Authors:** Kimiko Tomioka, Midori Shima, Keigo Saeki

**Affiliations:** ^1^ Nara Community Health Science Center, Nara Medical University, 88 Shijo-cho Kashihara, Nara 634-8524, Japan, naramed-u.ac.jp

**Keywords:** low back pain, prevalence, questionnaire, reliability, test–retest, validity

## Abstract

**Background:**

Low back pain (LBP) is one of the most prevalent pain conditions worldwide and a major contributor to disability. Although numerous epidemiological studies have investigated LBP, heterogeneity in pain definitions and assessment indicators has limited comparability across studies. A brief and reproducible questionnaire incorporating core LBP indicators commonly used in pain epidemiology may facilitate consistent assessment of LBP occurrence and impact in population‐based research.

**Methods:**

We proposed a brief low back pain questionnaire (B‐LBPQ) designed to assess the occurrence and characteristics of LBP in epidemiological studies. The B‐LBPQ consists of seven items covering current LBP, LBP during the past 12 months, lifetime experience of LBP, pain duration, and pain‐related interference with daily activities. A cross‐sectional validation study was conducted among 783 Japanese welfare workers aged 20–74 years (mean age: 41.9 years) undergoing routine health checkups. Construct validity was examined by comparing questionnaire‐based LBP classifications with scores on the Roland–Morris Disability Questionnaire (RDQ). Internal consistency was assessed using Cronbach’s alpha. Test–retest reliability over a 2‐week interval was evaluated among 595 participants using kappa statistics, prevalence‐adjusted bias‐adjusted kappa (PABAK), and weighted kappa coefficients.

**Results:**

Construct validity was supported by significantly higher RDQ scores among participants reporting LBP compared with those without LBP (all *p* < 0.001). For items with multiple response categories, significant differences and ordered trends in RDQ scores were observed across response categories (all *p* for trend < 0.001). Internal consistency was acceptable (Cronbach’s *α* = 0.87). Test–retest reliability ranged from substantial to almost perfect, with PABAK values of 0.70–0.85 for dichotomous items and weighted kappa coefficients of 0.65–0.81 for items with three or more categories.

**Conclusions:**

The B‐LBPQ demonstrated initial reliability and construct validity in Japanese welfare workers and may be useful for epidemiological assessment of LBP. However, further validation in diverse populations and settings is required.

## 1. Introduction

Low back pain (LBP) is a major global health problem and a leading cause of disability worldwide. Recent estimates from the Global Burden of Disease study indicate that more than 600 million people were affected by LBP in 2020, and this number is projected to increase substantially in the coming decades [1]. In addition, LBP imposes a considerable burden on individuals and society, including activity limitation, work absence, and economic costs [2, 3].

In epidemiological studies, clear and comparable definitions of LBP are essential for estimating prevalence and comparing findings across populations and settings. Previous efforts have emphasized the importance of standardizing definitions of LBP in prevalence studies to improve comparability across studies [4]. However, practical tools that balance clarity, comparability, and feasibility for large‐scale epidemiological use remain limited.

Several well‐established instruments are available for assessing disability or functional limitations among patients with LBP, such as the Oswestry Disability Index [5], the Roland–Morris Disability Questionnaire (RDQ) [6], and the Quebec Back Pain Disability Scale [7]. The Nordic Musculoskeletal Questionnaire (NMQ) [8] and its extended versions [9, 10] are widely used in occupational research. However, many of these instruments are relatively detailed and may impose a substantial burden on respondents, making them less suitable for large‐scale epidemiological studies. In addition, existing instruments are not always primarily designed to capture key epidemiological dimensions of LBP, such as current pain, past‐year prevalence, pain duration, and impact on daily activities, in a simple format. Therefore, there is a need for a brief and practical questionnaire that can assess essential epidemiological characteristics of LBP while maintaining feasibility for use in large populations.

In this study, we aimed to propose a brief LBP questionnaire (B‐LBPQ) for epidemiological assessment based on existing instruments and previous studies, with modifications to improve feasibility. Here, “brief” refers to a questionnaire with a limited number of items and low respondent burden, designed for use in large‐scale epidemiological studies. The B‐LBPQ focuses on key dimensions such as prevalence, duration, and pain‐related interference with daily activities. This approach is intended to complement, rather than replace, existing instruments by providing a feasible option for epidemiological studies.

## 2. Materials and Methods

### 2.1. Study Design and Data Source

This study employed a cross‐sectional design to examine the construct validity and reliability of a B‐LBPQ as a pain‐related epidemiological outcome measure. Data for the validity assessment were obtained from routine health checkups conducted by employers for employee health management. An opt‐out procedure was implemented to allow participants to decline participation. For the assessment of test–retest reliability, a follow‐up survey was conducted 2 weeks after the initial survey among participants who provided written informed consent. Data from this follow‐up survey were used exclusively for the evaluation of reproducibility. The study protocol was approved by the Ethics Committee of Nara Medical University (approval number 3673).

### 2.2. Study Population

Potential participants were 784 employees working at a social welfare corporation in Osaka Prefecture, Japan, with scheduled working hours of 30 h or more per week. In October 2024, 783 workers completed a self‐administered questionnaire and were included in the construct validity analysis. Two weeks later, 595 participants completed the B‐LBPQ again and were included in the test–retest reliability analysis. Participants included in the test–retest analysis were significantly older than those excluded from the analysis; however, no significant differences were observed with respect to sex or responses to LBP‐related questions (S1 Table).

### 2.3. B‐LBPQ

The B‐LBPQ was constructed to capture core epidemiological indicators of LBP for use in population‐based and occupational studies. Previous LBP studies were reviewed to identify relevant prevalence periods and other indicators, including a 1‐week prevalence, and these findings are summarized in S2 Table. The final B‐LBPQ included current pain, LBP during the past 12 months, lifetime history of LBP, pain duration, chronicity, and pain‐related interference with daily activities. Details of the selection process are provided in S3 Appendix. The initial item pool was generated based on established instruments and previous studies on LBP, including the Japanese Low Back Pain Evaluation Questionnaire [11], the Standardized Nordic Questionnaire [8], and relevant epidemiological and clinical literature [9, 10, 12–17]. Expert input was obtained from specialists in occupational LBP to review the relevance and feasibility of the candidate items. A preliminary version was pilot‐tested in approximately 30 individuals before the main study, and feedback was collected regarding clarity, response burden, and feasibility. Based on this feedback and further literature review, 1 week and monthly prevalence, which were identified as potential timeframes in previous studies, were not included in the final questionnaire in order to maintain brevity and reduce respondent burden. Current and past‐year LBP, together with lifetime history of LBP, were retained in the final questionnaire as core epidemiological indicators. Formal content validity testing was not conducted.

The final questionnaire consists of seven items: three assessing current LBP, three assessing LBP during the past 12 months, and one assessing lifetime history of LBP. Table 1 summarizes the questionnaire structure, including the questions, response formats, and the specific aspects of LBP assessed by each item, thereby clarifying how the questionnaire can be used for epidemiological classification, such as the identification of chronic LBP. The full questionnaire is provided in S4 Appendix. The original questionnaire was prepared and administered in Japanese, and the English version is provided for reporting purposes.

**TABLE 1:** Structure and epidemiological classification framework of the Brief Low Back Pain Questionnaire.

Questions		Response format	LBP assessment	
Q1	Do you have LBP today? If “No” is selected, then skip to Question 4.	Two options: Yes/No	Current LBP	Chronic LBP
Q2	How long have you had your current episode of LBP?	Three options: 1. < 4 weeks 2. 4 weeks to < 3 months 3. ≥ 3 months		

Q3	How much does your current LBP make it difficult for you to do your daily activities (e.g., work, school, regular tasks, household chores, etc.)?	Five options: 1. Not difficult 2. Mildly difficult 3. Moderately difficult 4. Considerably difficult 5. Extremely difficult		Problems with daily activities due to current LBP

Q4	Have you had LBP at any time during the past 12 months? If “No” is selected, then skip to Question 7.	Two options: Yes/No	Past‐year LBP	Past‐year LBP assessment based on duration of pain and activity restriction
Q5	What is the total length of time that you have had LBP during the past 12 months?	Four options: 1. < 8 days 2. 8 to < 30 days 3. ≥ 30 days, but not every day 4. Every day		
Q6	What is the total length of time that LBP has prevented you from doing your daily activities during the past 12 months?	Four options: 1. 0 days 2. 1–7 days 3. 8 to < 30 days 4. ≥ 30 days		

Q7	Have you ever had LBP?	Two options: Yes/No	Lifetime LBP	

*Note:* Low back pain was defined as pain or discomfort localized between the costal margin and the inferior gluteal folds, with or without leg pain. The original questionnaire was prepared and administered in Japanese. The English wording is provided for reporting purposes.

Abbreviation: LBP = low back pain.

Based on established definitions in pain research and guidelines [12–17], chronic LBP was defined as pain persisting for 3 months or longer. In this study, this definition was operationalized by combining information on current LBP and pain duration; participants who reported current LBP and pain persisting for 3 months or longer were classified as having chronic LBP. The duration categories for LBP during the past 12 months were based on the Standardized Nordic Questionnaire [8], which assesses cumulative symptom duration over the past year.

Pain‐related interference with daily activities was included as an indicator of pain impact, reflecting functional impact rather than pain intensity [8, 11, 13, 18]. Pain intensity was not assessed to reduce respondent and administrative burden and to maintain feasibility in epidemiological surveys. The item assessing pain‐related interference with daily activities was based on a previously validated Japanese questionnaire [11], which evaluates the impact of LBP on activities such as work or school, regular tasks, and household chores using a five‐point scale. This concept is also consistent with the Standardized Nordic Questionnaire [8], which assesses the impact of musculoskeletal symptoms on normal work. In this study, the timeframe was modified from “the last several days” [11] to “current” LBP to improve applicability in epidemiological research.

The English version presented in this study was translated by the authors for reporting purposes. The Japanese‐to‐English translation was reviewed by a native Japanese‐speaking bilingual reviewer, and the English version was proofread by a native English speaker. A formal cross‐cultural adaptation process, including back‐translation, cognitive debriefing with target‐language respondents, and validation in non‐Japanese populations, was not conducted.

### 2.4. RDQ

The RDQ [6] was used to assess disability due to LBP and is a widely used instrument for assessing functional limitation associated with LBP [19]. The Japanese version of the RDQ has demonstrated acceptable validity and reliability [20], supporting its use in Japanese‐speaking populations. Given that the present study was conducted in a working‐population setting rather than a clinical setting focusing on severe cases, the RDQ was considered appropriate because it is commonly used in epidemiological studies and is suitable for assessing disability in individuals with mild to moderate LBP, whereas the Oswestry Disability Index [5] is more commonly used in clinical settings, particularly among patients with more severe disability [19, 21]. The RDQ consists of 24 dichotomous items, with higher scores indicating greater functional impairment. In this study, RDQ scores were used as a related but conceptually distinct measure to examine the construct validity of the B‐LBPQ. We hypothesized that participants with LBP, as well as those with greater pain‐related interference with daily activities or longer pain duration, would have higher RDQ scores. This hypothesis was based on the assumption that greater impact of LBP would be associated with greater functional limitation.

### 2.5. Other Variables

Variables previously reported to be associated with LBP in pain epidemiological research were assessed, including age, sex, caregiver burden at home, lifestyle habits, physical workload, healthcare utilization for LBP, poor sleep, and poor mental health [15, 17, 22, 23]. Physical workload was assessed by the frequency of heavy handling at work. Heavy handling at work was assessed as an occupational exposure variable for examining known‐group validity [22] and was not part of the B‐LBPQ. Therefore, the psychometric properties of this exposure item were not evaluated in the present study. In healthcare and welfare settings, physical workload related to manual handling cannot be adequately defined by weight alone because it depends on multiple factors, such as posture, distance, twisting, frequency, and the level of assistance required. This approach is consistent with occupational health guidance on manual handling in healthcare and welfare work, including the Japanese guidelines for the prevention of LBP in the workplace [24] and ISO/TR 12296 [25]. For feasibility in the questionnaire‐based survey, manual handling of people or objects weighing approximately 5 kg or more was used as a practical reference threshold to facilitate consistent interpretation by respondents. This threshold was not intended to represent an ergonomic exposure limit. However, a large‐scale occupational study of workers in major industries in China reported that lifting loads weighing more than 5 kg was significantly associated with LBP [26]. Although this previous study [26] focused on lifting objects rather than manual handling of people, it supports the relevance of handling loads above approximately 5 kg as an occupational exposure indicator associated with LBP. Poor mental health was assessed using the Japanese version of the Kessler six‐item Psychological Distress Scale (K6) [27]; a higher score indicates a higher severity of mental health problems. Based on previous studies [27, 28], a score of < 5 was classified as no problem, 5–9 as mild, 10–12 as moderate, and > 12 as serious.

### 2.6. Statistical Analyses

Construct validity was evaluated by examining convergent validity based on predefined hypotheses regarding the direction of associations between B‐LBPQ measures and RDQ scores. Specifically, we hypothesized that participants with LBP, as well as those with greater pain classification or longer duration, would have higher RDQ scores, reflecting greater LBP‐related disability. Because RDQ scores were not normally distributed (S5 Figure), nonparametric tests were applied. For binary items, the Mann–Whitney *U* test was used, and for items with three or more categories, the Kruskal–Wallis test followed by the Bonferroni correction was performed. Trend associations were examined using the Jonckheere–Terpstra test. The hypotheses were considered supported if the observed associations were consistent with the expected direction and statistically significant. Discriminant validity was not assessed because no prespecified external measures expected to be weakly or unrelated to LBP were included. Structural validity, including exploratory or confirmatory factor analysis, was also not assessed because the B‐LBPQ was designed as a brief epidemiological questionnaire assessing several key dimensions of LBP rather than as a unidimensional scale.

Internal consistency was assessed using Cronbach’s alpha. An alpha value of ≥ 0.70 was considered to indicate acceptable internal consistency reliability based on commonly accepted criteria [29]. Because the questionnaire uses a skip pattern and was designed to assess several key epidemiological dimensions of LBP rather than to function as a unidimensional scale, Cronbach’s alpha should be interpreted with caution and was reported for descriptive purposes only; it was not intended to demonstrate the unidimensionality or structural validity of the questionnaire. Test–retest reliability of categorical items was evaluated using kappa statistics [30]. Kappa coefficient was characterized as follows [31]: poor, 0.00–0.20; fair, 0.21–0.40; moderate, 0.41–0.60; substantial, 0.61–0.80; almost perfect, 0.81–1.00. Given the known influence of prevalence and bias on kappa coefficients [32], prevalence‐adjusted bias‐adjusted kappa (PABAK) was additionally calculated for dichotomous items [33]. Weighted kappa coefficients with quadratic weights were used for items with three or more response categories [34]. In this study, we focused on evaluating fundamental psychometric properties, including test–retest reliability, internal consistency, and construct validity, which are considered essential for the initial validation of a questionnaire.

To examine whether pain prevalence assessed by the B‐LBPQ showed expected associations with established pain‐related factors, current LBP and chronic LBP were analyzed separately as binary outcomes. Although these two outcomes were correlated within individuals, chronic LBP was operationally defined using information on current LBP and pain duration; therefore, the two outcomes were not treated as independent repeated measurements in a joint model. For each outcome, multivariable modified Poisson regression models with robust standard errors were used to estimate adjusted prevalence ratios (APRs) and 95% confidence intervals (CIs). Because the outcomes were relatively common, prevalence ratios were considered more interpretable than odds ratios, which can overestimate the magnitude of association when outcomes are common. The models were adjusted for sex, age, caregiver burden at home, and lifestyle factors. Caregiver burden at home included the presence of preschool children and cohabitation with a family member requiring care, and lifestyle factors included exercise habits, smoking habits, and drinking habits. These variables were selected because they are known or suspected to be associated with LBP [15, 17, 22, 23, 26].

Missing data were handled as follows. Among the 783 participants, 16 participants (2.0%) had at least one missing value among the covariates included in the multivariable analyses. In total, 31 covariate values were missing, with the number of missing values per variable ranging from 0 to 7 (0.0%–0.9% of participants). Missing covariate values were imputed using multiple imputation by chained equations, generating five imputed datasets. The imputation model included sex, age, the presence of preschool children, cohabitation with a family member requiring care, exercise habits, smoking habits, alcohol drinking habits, and RDQ score. RDQ scores were available for all participants. Estimates from the five imputed datasets were combined using Rubin’s rules. For questionnaire items assessing LBP, no imputation was performed, and participants with missing responses were excluded from the corresponding analyses using a complete‐case approach. The number of participants included in each analysis is reported in the corresponding tables. In addition, the distribution of responses for each covariate, including the number and percentage of missing values, is presented in Table 2.

**TABLE 2:** Demographic characteristics of study participants (*N* = 783), including the distribution of covariates and missing values.

		*N* (%)	
Sex	Male	242	(30.9)
	Female	541	(69.1)
	Missing	0	(0.0)

Age	20–29	193	(24.6)
	30–39	134	(17.1)
	40–49	195	(24.9)
	50–59	185	(23.6)
	≥ 60	69	(8.8)
	Missing	7	(0.9)

Preschool children	Present	95	(12.1)
	Absent	685	(87.5)
	Missing	3	(0.4)

Family member requiring care	Present	114	(14.6)
	Absent	663	(84.7)
	Missing	6	(0.8)

Exercise habits	Present	157	(20.1)
	Absent	619	(79.1)
	Missing	7	(0.9)

Smoking habits	Current smoker	130	(16.6)
	Former smoker	148	(18.9)
	Never smoker	501	(64.0)
	Missing	4	(0.5)

Alcohol drinking habits	Almost every day	141	(18.0)
	3–5 days per week	51	(6.5)
	1–2 days per week	126	(16.1)
	< 1 day per week	461	(58.9)
	Missing	4	(0.5)

*Note:* Data are presented as *n* (%). Missing values are shown for each variable. Percentages are calculated based on the total number of participants (*N* = 783). Exercise habits: defined as engaging in regular exercise or sports for at least 1 year, with a frequency of at least twice per week and a duration of at least 30 min per session. Smoking habits: categorized as current smoker, former smoker, or never smoker. Alcohol drinking habits: categorized based on frequency of alcohol consumption as almost every day, 3–5 days per week, 1–2 days per week, or less than 1 day per week. Sixteen participants (2.0%) had at least one missing value among the covariates included in the multivariable analyses. In total, 31 covariate values were missing, and the number of missing values per variable ranged from 0 to 7 (0.0%–0.9% of participants).

All statistical analyses were performed using the IBM SPSS Statistics Ver. 29 for Windows (Armonk, New York, US), and the significance level was set at 0.05 (two‐tailed test).

### 2.7. Ethical Approval

This study was conducted in accordance with the Declaration of Helsinki and was approved by the Ethics Committee of Nara Medical University (approval number 3673). In addition, permission to conduct this study was obtained from the Occupational Safety and Health Committee of the corporation where the study was conducted. For the validity study, we produced an opt‐out document to give participants the opportunity to decline. For the test–retest reliability study, participants gave written informed consent prior to their inclusion in the study.

## 3. Results

### 3.1. Characteristics of Study Participants

Among the 783 study participants, the median [25%ile, 75%ile] of the RDQ score was 0.0 [0.0, 2.0], with 59.6% of participants having an RDQ score of 0 (S5 Figure). Regarding the prevalence of LBP assessed by the B‐LBPQ, 16.3% suffered from chronic LBP, point prevalence of current LBP was 36.8%, past‐year LBP prevalence was 75.6%, and lifetime LBP prevalence was 81.6%. The average age was 41.9 years, and 69.1% were women. Among the participants, 12.1% had preschool children, 14.6% lived with a family member requiring care, and 20.1% reported regular exercise habits (Table 2).

### 3.2. Construct Validity (Table 3)

For questions 1, 4, and 7, and the presence or absence of chronic LBP, which had two categories, there were significant differences between the two groups (*p* values < 0.001 in all items). For questions 2, 3, 5, and 6, and the reclassified LBP assessments, which had three or more categories, RDQ scores differed significantly across categories, and significant ordered trends were observed (*p* for trend < 0.001 in all analyses). For current LBP duration (Question 2), however, the median RDQ score was significantly higher in the 4 weeks to < 3‐month category than in the ≥ 3‐month category after the Bonferroni correction. Thus, although the overall findings were broadly consistent with our predefined hypotheses regarding construct validity, RDQ scores did not increase monotonically across all categories of current LBP duration.

**TABLE 3:** Construct validity assessment of the B‐LBPQ: median and IQR of participants’ RDQ score.

	*N*	RDQ score	*p* ^a^	Multiple comparisons by Bonferroni correction		*p* for trend^b^
		Median [IQR]				
*LBP today (current LBP)*						
Q1 Presence or absence of current LBP			^∗∗c^			
1. Absent	493	0.0 [0.0–0.0]				
2. Present	287	2.0 [1.0–5.0]				
Missing	3					
Q2 Duration of current LBP			^∗∗d^			^∗∗^
1. Less than 4 weeks	118	1.0 [0.0–2.0]		1 vs 2^∗∗^		
2. 4 weeks to < 3 months	37	4.0 [1.0–8.0]		1 vs 3^∗∗^		
3. ≥ 3 months	126	3.0 [1.0–6.0]		2 vs 3^∗^		
Missing	6					
Q3 Problems with daily activities due to current LBP			^∗∗d^			^∗∗^
1. Not difficult	186	1.0 [0.0–3.0]		1 vs 2^∗∗^	2 vs 3 NS	
2. Mildly difficult	78	4.5 [2.0–9.0]		1 vs 3^∗^	2 vs 4^∗^	
3. Moderately difficult	9	8.0 [5.0–9.0]		1 vs 4^∗∗^	3 vs 4 NS	
4. Considerably/extremely difficult	8	13.0 [12.0–15.0]				
Missing	6					

*LBP during the past 12 months (past-year LBP)*						
Q4 Presence or absence of past‐year LBP			^∗∗c^			
1. Absent	189	0.0 [0.0–0.0]				
2. Present	587	1.0 [0.0–2.0]				
Missing	7					
Q5 Duration of past‐year LBP			^∗∗d^			^∗∗^
1. < 8 days	211	0.0 [0.0–0.0]		1 vs 2^∗∗^	2 vs 3^∗∗^	
2. 8 to < 30 days	151	0.0 [0.0–2.0]		1 vs 3^∗∗^	2 vs 4^∗∗^	
3. ≥ 30 days, but not every day	159	2.0 [1.0–5.0]		1 vs 4^∗∗^	3 vs 4 NS	
4. Every day	66	3.0 [2.0–7.0]				
Q6 Duration of disruption to daily activities due to past‐year LBP			^∗∗d^			^∗∗^
1. 0 days	332	0.0 [0.0–1.5]		1 vs 2^∗∗^	2 vs 3^∗∗^	
2. 1–7 days	173	1.0 [0.0–3.0]		1 vs 3^∗∗^	2 vs 4^∗∗^	
3. 8 to < 30 days	49	4.0 [1.0–9.0]		1 vs 4^∗∗^	3 vs 4 NS	
4. ≥ 30 days	29	9.0 [4.0–13.0]				
Missing	4					

*Lifetime experience of LBP (lifetime LBP)*						
Q7 Presence or absence of lifetime LBP			^∗∗c^			
1. Absent	144	0.0 [0.0–0.0]				
2. Present	637	0.0 [0.0–2.0]				
Missing	2					

*Current LBP assessment using questions 1 & 2*						
Presence or absence of chronic LBP			^∗∗c^			
1. No chronic LBP	648	0.0 [0.0–1.0]				
2. Chronic LBP	126	3.0 [1.0–6.0]				
Missing	9					
Current LBP assessment based on pain duration			^∗∗d^			^∗∗^
1. No LBP	493	0.0 [0.0–0.0]		1 vs 2^∗∗^		
2. Acute/subacute LBP	155	2.0 [0.0–4.0]		1 vs 3^∗∗^		
3. Chronic LBP	126	3.0 [1.0–6.0]		2 vs 3^∗∗^		
Missing	9					

*Current LBP assessment using questions 1 & 3 (reclassified)*			^∗∗d^			^∗∗^
1. No LBP	493	0.0 [0.0–0.0]		1 vs 2^∗∗^	2 vs 3^∗∗^	
2. LBP without daily activity difficulty	186	1.0 [0.0–3.0]		1 vs 3^∗∗^	2 vs 4^∗∗^	
3. LBP with mild daily activity difficulty	78	4.5 [2.0–9.0]		1 vs 4^∗∗^	3 vs 4 NS	
4. LBP with moderate or greater daily activity difficulty	17	11.0 [8.0–13.0]				
Missing	9					

*Past-year LBP assessment based on pain duration and activity restriction* ^e^			^∗∗d^			^∗∗^
1. No past‐year LBP	189	0.0 [0.0–0.0]		1 vs 2^∗^	2 vs 4^∗∗^	
2. Past‐year LBP < 30 days without activity restriction	232	0.0 [0.0–1.0]		1 vs 3^∗∗^	2 vs 5^∗∗^	
3. Past‐year LBP < 30 days with activity restriction	129	0.0 [0.0–2.0]		1 vs 4^∗∗^	3 vs 4^∗^	
4. Past‐year LBP ≥ 30 days without activity restriction	100	2.0 [0.0–2.5]		1 vs 5^∗∗^	3 vs 5^∗∗^	
5. Past‐year LBP ≥ 30 days with activity restriction	122	4.0 [1.0–9.0]		2 vs 3^∗^	4 vs 5^∗∗^	
Missing	11					

*Note:* Those with missing data on each question were excluded from the analysis of each question. Questions 2 and 3 were limited to those with current LBP. Questions 5 and 6 were limited to those with past‐year LBP.

Abbreviations: B‐LBPQ = Brief Low Back Pain Questionnaire, IQR = interquartile range, LBP = low back pain, NS = not significant, RDQ = Roland–Morris Disability Questionnaire.

^∗^ *p* < 0.05.

^∗∗^ *p* < 0.001.

^a^ *p* value based on nonparametric test.

^b^Jonckheere–Terpstra test.

^c^Mann–Whitney test.

^d^Kruskal–Wallis test.

^e^Using questions 4–6.

### 3.3. Reliability (Table 4 and S6 and S7 Appendices)

The B‐LBPQ showed Cronbach’s alpha coefficient to be 0.87, indicating an acceptable internal consistency reliability. For dichotomous items, kappa coefficients ranged from 0.68 to 0.73, indicating substantial agreement: presence or absence of chronic LBP had the lowest coefficient (*κ* [95% CI] = 0.68 [0.60–0.76]) and presence or absence of past‐year LBP had the highest coefficient (*κ* [95% CI] = 0.73 [0.66–0.80]). When PABAK was used to assess the test–retest reliability, PABAK coefficients ranged from 0.70 to 0.85, indicating a substantial‐to‐almost perfect agreement: presence or absence of current LBP had the lowest coefficient (PABAK = 0.70) and lifetime experience of LBP had the highest coefficient (PABAK = 0.85). For items with three or more categories for which reliability was assessed using weighted kappa coefficients, five of the six items had substantial agreement, and one item had almost perfect agreement, with weighted kappa coefficients ranging from 0.65 to 0.81: the item on problems with daily activities due to current LBP had the lowest coefficient (weighted *κ* [95% CI] = 0.65 [0.51–0.78]) and past‐year LBP assessment using questions 4–6 had the highest coefficient (weighted *κ* [95% CI] = 0.81 [0.78–0.85]).

**TABLE 4:** Two‐week test–retest reliability of the B‐LBPQ.

	*N*	Dichotomous items				Items with ≥ 3 categories
		*κ* (95% CI)	PABAK	PI	BI	Weighted *κ* (95% CI)
LBP today (current LBP)						
Q1 Presence or absence of current LBP	595	0.69 (0.63–0.75)	0.70	−0.22	0.03	
Q2 Duration of LBP	182					0.70 (0.61–0.80)
Q3 Problems with daily activities due to current LBP	183					0.65 (0.51–0.78)
LBP during the past 12 months (past‐year LBP)						
Q4 Presence or absence of past‐year LBP	586	0.73 (0.66–0.80)	0.81	0.55	0.04	
Q5 Duration of past‐year LBP	427					0.78 (0.73–0.84)
Q6 Duration of disruption to daily activities due to past‐year LBP	424					0.73 (0.66–0.80)
Lifetime experience of LBP (lifetime LBP)						
Q7 Presence or absence of lifetime LBP	594	0.73 (0.66–0.81)	0.85	0.66	0.03	
LBP assessment using questions 1 & 2						
Presence or absence of chronic LBP	586	0.68 (0.60–0.76)	0.82	−0.65	0.01	
LBP assessment based on pain duration	586					0.75 (0.70–0.80)
Past‐year LBP assessment using questions 4 to 6: 5 categories	580					0.81 (0.78–0.85)

*Note:* Those with missing data on each question were excluded from the analysis of each question. Questions 2 and 3 were limited to those with current LBP. Questions 5 and 6 were limited to those with past‐year LBP.

Abbreviations: *κ* = kappa coefficient, BI = bias index, B‐LBPQ = Brief Low Back Pain Questionnaire, CI = confidence interval, LBP = low back pain, PABAK = prevalence‐adjusted bias‐adjusted kappa, PI = prevalence index.

### 3.4. Association Between LBP Assessed by the B‐LBPQ and Factors Associated With LBP (Table 5)

In, the separate multivariable modified Poisson regression models, higher levels of established LBP‐related factors were generally associated with a higher prevalence of current LBP and chronic LBP. These associations showed dose–response patterns for several factors. These results supported the known‐group validity of LBP assessed with the B‐LBPQ.

**TABLE 5:** Association between LBP assessed by the B‐LBPQ and known related factors.

	Current LBP				Chronic LBP			
	*N*	%	APR (95% CI)	*p* value	*N*	%	APR (95% CI)	*p* value
Frequency of heavy handling at work								
Little or none	170	24.1	1.00		168	10.1	1.00	
Several times per month	169	32.0	1.40 (0.99–2.00)	0.058	167	13.8	1.38 (0.76–2.53)	0.295
Several times per week	94	34.0	**1.53 (1.04–2.25)**	**0.030**	93	17.2	1.73 (0.92–3.26)	0.087
Daily (< 2 h per day)	285	43.5	**1.97 (1.45–2.66)**	**< 0.001**	285	19.3	**2.10 (1.24–3.54)**	**0.006**
Daily (≥ 2 h per day)	58	58.6	**2.72 (1.92–3.87)**	**< 0.001**	57	24.6	**2.80 (1.44–5.46)**	**0.003**
			*p* for trend < 0.001				*p* for trend < 0.001	
Treatment for LBP in the past 12 months								
None	419	21.2	1.00		417	7.4	1.00	
Several times per year	191	44.5	**2.07 (1.62–2.64)**	**< 0.001**	190	18.4	**2.42 (1.54–3.80)**	**< 0.001**
Several times per month	102	60.8	**2.92 (2.28–3.75)**	**< 0.001**	102	32.4	**4.32 (2.73–6.85)**	**< 0.001**
Almost every week	63	77.8	**3.61 (2.85–4.58)**	**< 0.001**	60	43.3	**5.56 (3.50–8.83)**	**< 0.001**
			*p* for trend < 0.001				*p* for trend < 0.001	
Sleep restfulness								
Very restful	81	19.8	1.00		81	7.4	1.00	
Restful	397	31.5	**1.59 (1.001–2.53)**	**0.049**	395	13.9	1.79 (0.78–4.10)	0.171
Restless	268	46.6	**2.29 (1.44–3.63)**	**< 0.001**	264	20.8	**2.52 (1.10–5.79)**	**0.030**
Very restless	31	64.5	**3.20 (1.91–5.36)**	**< 0.001**	31	29.0	**3.54 (1.34–9.31)**	**0.010**
			*p* for trend < 0.001				*p* for trend < 0.001	
Psychological distress (K6 score)								
None (< 5)	502	31.7	1.00		500	13.6	1.00	
Mild (5–9)	149	47.0	**1.44 (1.16–1.78)**	**0.001**	147	19.0	1.31 (0.88–1.95)	0.182
Moderate (10–12)	53	43.4	1.40 (0.99–1.99)	0.059	52	17.3	1.31 (0.69–2.46)	0.409
Serious (> 12)	76	46.1	**1.46 (1.11–1.91)**	**0.007**	75	28.0	**2.02 (1.30–3.13)**	**0.002**
			*p* for trend < 0.001				*p* for trend = 0.002	

*Note:* APRs and 95% CIs were estimated using separate multivariable modified Poisson regression models with robust standard errors for current LBP and chronic LBP. Results in bold indicate *p* < 0.05. The models were adjusted for sex, age, caregiver burden at home, and lifestyle factors. Caregiver burden at home included the presence of preschool children and cohabitation with a family member requiring care, and lifestyle factors included exercise habits, smoking habits, and drinking habits. Missing covariate values were imputed using multiple imputation by chained equations with five imputed datasets, and the estimates were combined using Rubin’s rules. Heavy handling at work was defined as manual handling of people or objects weighing approximately 5 kg or more. The threshold of approximately 5 kg was used as a practical reference to help respondents interpret the frequency of manual handling of people or objects; it was not intended to represent an ergonomic exposure limit.

Abbreviations: APR = adjusted prevalence ratio, B‐LBPQ = Brief Low Back Pain Questionnaire, CI = confidence interval, LBP = low back pain, K6 = the Kessler six‐item Psychological Distress Scale.

## 4. Discussion

In this study, we proposed and initially validated a B‐LBPQ designed for epidemiological assessment of LBP occurrence and impact. The questionnaire demonstrated acceptable construct validity and reliability in a working population, supporting its use as a practical tool for pain‐related research requiring concise and reproducible measurement.

### 4.1. Pain‐Related Construct Validity

Construct validity of the B‐LBPQ was supported by several complementary findings. First, participants reporting LBP on the questionnaire consistently showed higher RDQ scores than those without LBP. Although the RDQ primarily assesses functional limitation rather than pain intensity per se, functional impairment is a well‐established consequence of pain and is conceptually linked to overall pain occurrence and impact [6, 35]. Therefore, higher RDQ scores among individuals reporting LBP provide evidence that the B‐LBPQ captures clinically meaningful pain‐related experiences. Second, for items with multiple response categories, significant ordered trends in RDQ scores were observed across response categories. However, for current LBP duration, the median RDQ score was significantly higher in the 4 weeks to < 3‐month category than in the ≥ 3‐month category after the Bonferroni correction; therefore, RDQ scores did not increase monotonically across all categories of current LBP duration. In contrast, greater pain–related interference with daily activities and more burdensome past‐year LBP classifications were generally associated with higher RDQ scores. These findings broadly support the plausibility that the B‐LBPQ reflects gradations in pain‐related impact rather than merely dichotomous pain presence. Third, known‐groups validity was supported by the observed associations between LBP assessed by the B‐LBPQ and factors consistently reported in the pain literature, including heavy physical workload [36], healthcare utilization for LBP [37], poor sleep [38], and poor mental health [39]. The presence of graded, dose–response relationships for both current and chronic LBP further supports the construct validity of the questionnaire as a pain‐related epidemiological outcome measure.

### 4.2. Reliability as an Epidemiological Pain Outcome

The B‐LBPQ showed acceptable internal consistency and substantial to almost perfect test–retest reliability. While internal consistency estimates should be interpreted cautiously due to the conditional structure of the questionnaire, test–retest reliability over a 2‐week interval demonstrated good reproducibility across both dichotomous and multicategory items. The use of PABA addressed known limitations of conventional kappa statistics in high‐ or low‐prevalence conditions and provided a more robust assessment of agreement [32, 33]. The 2‐week interval may have allowed for some true change in pain status, particularly among individuals with acute LBP [40]; however, this interval reduces the likelihood of recall effects and aligns with reliability testing intervals used in other musculoskeletal and pain‐related questionnaires [11, 20, 41, 42]. Taken together, these findings indicate that the B‐LBPQ provides stable estimates of pain occurrence and impact suitable for epidemiological research.

### 4.3. Positioning of Pain Indicators and Omission of Pain Intensity

The B‐LBPQ intentionally focuses on pain occurrence, duration, chronicity, and activity interference rather than pain intensity. Although pain intensity measures [43] such as visual analogue scale (VAS) and numerical rating scale are widely used in clinical settings, their inclusion in large‐scale epidemiological surveys may increase respondent burden and susceptibility to recall bias, particularly when longer reference periods are employed. In pain epidemiology, pain duration and pain‐related interference with daily activities are frequently used as key indicators of pain impact and have demonstrated strong associations with functional outcomes, healthcare utilization, and quality of life [35, 44]. Moreover, prior research has shown that pain intensity and functional impact are related but distinct constructs, underscoring the importance of assessing dimensions beyond intensity alone [13]. By integrating pain duration and activity interference across different time frames, the B‐LBPQ provides an alternative approach to capturing pain occurrence and impact that complements intensity‐based measures. This design choice does not imply that pain intensity is unimportant but rather reflects a pragmatic balance between epidemiological feasibility and measurement burden. The present findings suggest that this approach yields meaningful differentiation among individuals with varying levels of pain‐related impact.

### 4.4. Comparison With Existing Instruments

Existing instruments such as the RDQ [6], Oswestry Disability Index [5], and the NMQ [8] have been widely used in LBP research, each serving different purposes. Disability‐focused instruments are well suited for evaluating functional outcomes among patients [5, 6], whereas the NMQ has been primarily applied in occupational health surveillance [8]. The B‐LBPQ differs from these instruments by integrating core pain epidemiological indicators—occurrence, duration, chronicity, and activity interference—within a brief format designed for use in population‐based and occupational surveys. Rather than replacing detailed clinical or disability assessments, the B‐LBPQ is intended to complement them by providing a consistent approach to measuring pain‐related characteristics in large‐scale studies. This positioning is consistent with the consensus approach proposed by Dionne et al. [4], which emphasized the need for standardized definitions of LBP in prevalence studies to improve comparability across populations and settings. By incorporating clearly defined epidemiological indicators within a brief format, the B‐LBPQ may support more consistent assessment and reporting of LBP in large‐scale epidemiological research.

### 4.5. Strengths and Limitations

This study has several strengths. The large sample size enabled a robust evaluation of the validity and reliability of the B‐LBPQ. Construct validity was examined using complementary approaches, including comparisons with a related functional measure and associations with established pain‐related factors. In addition, the questionnaire was designed to capture key epidemiological indicators of LBP while minimizing respondent burden, thereby prioritizing feasibility and reproducibility for large‐scale surveys.

Several limitations should be acknowledged. First, the 2‐week test–retest interval may have allowed true changes in LBP status, particularly among individuals with acute symptoms; therefore, disagreement between test and retest responses may reflect true change, measurement error, or both. For example, a previous study reported that 57.1% of patients with acute LBP recovered within 2 weeks, defined as an RDQ score < 4 and resolution of pain [40]. Conversely, a study of patients with knee disorders found no significant differences in test–retest reliability between 2 day and 2‐week intervals [45]. Although some change in symptoms over 2 weeks cannot be ruled out, this interval may reduce recall‐related bias and may therefore be advantageous for assessing reproducibility. Second, the study population consisted of Japanese welfare workers undergoing routine health checkups, which may introduce selection bias and limit the generalizability of the findings. Although we adjusted for potential confounders such as age and sex, participants who attend health checkups may be systematically healthier or more health‐conscious than the general population. In addition, because participants were recruited from a single occupational sector with a relatively high proportion of women, the findings may not be generalizable to other occupational groups, populations with different sex distributions, or the general population. Moreover, the questionnaire was tested only in a working population and has not been validated in other age or population groups, such as children and adolescents, adults aged over 65 years, or people with cognitive disorders. Third, although an English version of the questionnaire was provided, the questionnaire was originally prepared and administered in Japanese. The English version was translated by the authors, reviewed by a bilingual native Japanese speaker, and proofread by a native English speaker; however, a formal cross‐cultural adaptation process, including back‐translation, cognitive debriefing, and validation in non‐Japanese populations, was not performed. Therefore, caution is warranted when applying the questionnaire in different cultural or linguistic settings, and further validation is needed in other cultural environments. Fourth, the B‐LBPQ does not include a direct measure of pain intensity, such as a VAS [43]. This was an intentional design choice to maintain the brevity and feasibility of the instrument for large‐scale epidemiological use. The B‐LBPQ was designed to assess key epidemiological dimensions of LBP, including prevalence, duration, and pain‐related interference with daily activities, rather than to serve as a comprehensive clinical pain assessment tool. Although the VAS is widely used to assess pain intensity [43], its inclusion may increase respondent burden and administrative complexity. Instead, LBP‐related disability was assessed using the RDQ, which reflects the impact of LBP on daily functioning. Future studies may incorporate complementary measures of pain intensity depending on the research purpose. Fifth, because the B‐LBPQ is a symptom‐based epidemiological instrument, we did not collect clinical diagnoses or imaging findings and therefore could not account for specific etiologies of LBP. Future studies should examine whether its measurement properties differ in clinically characterized samples. Sixth, this study evaluated only a limited number of psychometric properties. Although test–retest reliability, internal consistency, and construct validity, including convergent validity and known‐groups validity, were examined, other important measurement properties, such as structural validity, measurement error, criterion validity, responsiveness, and interpretability, were not assessed. Because the B‐LBPQ was designed for large‐scale epidemiological assessment rather than clinical decision‐making, future studies should further examine structural validity, measurement error, and interpretability in different populations and survey settings. Responsiveness may also be evaluated in future longitudinal studies if the B‐LBPQ is used to assess changes in LBP status over time. In addition, we did not define explicit cut‐off criteria for hypothesis testing in the assessment of construct validity, and statistical significance and consistency with expected directions were used as the basis for interpretation. This exploratory approach may limit the rigor of the validation and should be refined in future studies. Seventh, several limitations relate to the assessment of LBP duration and temporal constructs. Although chronic LBP was defined based on established criteria, namely, pain lasting ≥ 3 months [12–17], its identification in this study required the presence of current pain. Therefore, chronic LBP could not be identified among participants without current pain, even if they had experienced prolonged symptoms in the past. Furthermore, the duration categories for LBP during the past 12 months were based on the Standardized Nordic Questionnaire [8] and did not include a specific ≥ 3‐month category. In addition, although the questionnaire includes items related to different timeframes of LBP, such as current and past‐year LBP, the validity of these temporal constructs was not fully examined. These limitations reflect a trade‐off between consistency with established epidemiological instruments and simplicity. Further research is needed to investigate the relationships and validity of these temporal dimensions. Eighth, although no significant differences were observed in sex or LBP‐related responses, participants included in the test–retest analysis were significantly older than those excluded. Therefore, the possibility of residual bias due to loss to follow‐up cannot be excluded. Finally, the item assessing LBP‐related interference with daily activities was adapted from existing multi‐item instruments into a single‐item measure to improve feasibility and reduce respondent burden. However, this simplification may limit the ability to capture specific domains of functional impairment, particularly in distinguishing between different types of daily activities, and may reduce measurement precision compared with multi‐item scales. Further validation is needed to examine the performance of this item in different populations and settings.

### 4.6. Implications for Pain Research

Despite these limitations, the B‐LBPQ offers a concise and reproducible tool for assessing LBP occurrence and impact in epidemiological research. Its focus on pain duration, chronicity, and activity interference aligns with key constructs in pain research and may facilitate more consistent reporting across studies. With further validation in diverse populations and languages, the B‐LBPQ may support more consistent assessment of LBP‐related pain outcomes in population‐based and occupational research settings.

## 5. Conclusions

This study proposed and initially validated a brief questionnaire for the epidemiological assessment of LBP in a population of Japanese welfare workers. The questionnaire may be useful for assessing the occurrence and characteristics of LBP in similar populations. However, as this study was conducted in a specific occupational group and evaluated a limited range of psychometric properties, further research is required to validate the questionnaire in more diverse populations and cultural contexts and to comprehensively assess its measurement properties. Therefore, the questionnaire should be considered a preliminary tool that may contribute to future epidemiological research on LBP.

## Author Contributions

Kimiko Tomioka contributed to conceptualization, formal analysis, investigation, methodology, resources, validation, visualization, and writing of the original draft. Keigo Saeki contributed to formal analysis, methodology, project administration, and writing through review and editing. Midori Shima contributed to supervision and writing through review and editing. All authors contributed to data curation and funding acquisition, and all authors approved the final manuscript.

## Funding

This study was supported by the Center Research Expense of Nara Medical University (no grant number).

## Conflicts of Interest

Kimiko Tomioka is employed as a contracted occupational physician for the Osaka Welfare Foundation, a social welfare corporation, from which the data used in this study were obtained. The data were used with the corporation’s approval and were anonymized prior to analysis. This does not alter our adherence to the policies of Pain Research and Management on sharing data and materials. The other authors declare no conflicts of interest.

## Supporting Information

Additional supporting information can be found online in the Supporting Information section.

## Supporting information


**Supporting Information 1** S1 Table: Comparison between participants included in and those excluded from the test–retest reliability study. S2 Table: List of previous studies on low back pain. S3 Appendix: Details regarding the selection process for indicators for the Brief Low Back Pain Questionnaire (B‐LBPQ). S4 Appendix: The translated version of the Brief Low Back Pain Questionnaire. S5 Figure: Distribution of RDQ scores among study participants. S6 Appendix: Distribution of responses in the test and retest: Questions 1–7. S7 Appendix: Distribution of responses in the test and retest: reclassification.

**Supporting Information 2** STROBE_Checklist_cross_sectional_B‐LBPQ.

## Data Availability

The data that support the findings of this study are available from the corresponding author upon reasonable request.
