# Supplementary material for: Proposal and Initial Validation of a Brief Questionnaire for Epidemiological Assessment of Low Back Pain
Source: Pain Res Manag. 2026 Aug 25;2026:6203306. doi: 10.1155/prm/6203306 (PMC13504453; doi:10.1155/prm/6203306)
Supplement: Supplementary file 1 [file PRM-2026-6203306-s002.pdf]

S1 Table: Comparison between participants included in and those excluded from the test-retest reliability study.

			The test-retest reliability study		
			Included (n = 595)	Excluded (n = 188)	*P*-value
			N (%)	N (%)	
Age, mean ± standard deviation			42.6 ± 12.8	39.8 ± 13.2	0.009[a]
Sex, male			191 (32.1)	51 (27.1)	0.206[b]
**LBP today (current LBP)**					
	Q1	Presence or absence of current LBP			
	1	Absence	372 (62.5)	121 (65.4)	0.487[b]
	2	Presence	223 (37.5)	64 (34.6)	
	Q2	Duration of current LBP			
	1	Less than 4 weeks	94 (43.1)	24 (38.1)	0.136[b]
	2	4 weeks to < 3 months	24 (11.0)	13 (20.6)	
	3	≥ 3 months	100 (45.9)	26 (41.3)	
	Q3	Problems with daily activities due to current LBP			
	1	Not difficult	142 (65.1)	44 (69.8)	0.518[c]
	2	Mildly difficult	64 (29.4)	14 (22.2)	
	3	Moderately difficult	7 (3.2)	2 (3.2)	
	4	Considerably/extremely difficult	5 (2.3)	3 (4.8)	
**LBP during the past 12 months (past-year LBP)**					
	Q4	Presence or absence of past-year LBP			
	1	Absent	145 (24.5)	44 (23.8)	0.845[b]
	2	Present	446 (75.5)	141 (76.2)	
	Q5	Duration of past-year LBP			
	1	< 8 days	159 (35.7)	52 (36.9)	0.972[b]
	2	8 to < 30 days	114 (25.6)	37 (26.2)	
	3	≥ 30 days, but not every day	123 (27.6)	36 (25.5)	
	4	Every day	50 (11.2)	16 (11.3)	
	Q6	Duration of disruption to daily activities due to past-year LBP			
	1	0 days	239 (54.1)	93 (66.0)	0.096[b]
	2	1–7 days	139 (31.4)	34 (24.1)	
	3	8 to < 30 days	40 (9.0)	9 (6.4)	
	4	≥ 30 days	24 (5.4)	5 (3.5)	
**Lifetime experience of LBP (lifetime LBP)**					
	Q7	Presence or absence of lifetime LBP			
	1	Absence	108 (18.2)	36 (19.4)	0.745[b]
	2	Presence	487 (81.8)	150 (80.6)	

Note: Unless otherwise stated, data are given in n (%). Those with missing data on each question were excluded from the analysis of each question. Questions 2 and 3 were limited to those with current LBP. Questions 5 and 6 were limited to those with past-year LBP. [a]T-test, [b]Chi-squared test, [c]Fisher's exact test. Abbreviations: LBP = low back pain.

S2 Table: List of previous studies on low back pain.

Authors, year of publication [Ref No.]	Country	Subjects	Prevalence period					Pain duration	Chronicity	Interference with daily activities
			Point	One week	One month	One year	Lifetime			
Almansour AH, 2024 [1]	Saudi Arabia	School teachers		✓		✓		✓		✓
Chen C, 2024 [2]	Worldwide	Professional drivers		✓	✓	✓				
Farahbakhsh F, 2018 [3]	Worldwide	Athletes	✓			✓	✓			
Hoy D, 2012 [4]	Worldwide	General population	✓		✓	✓	✓			
Jia N, 2022 [5]	China	Occupational population				✓				
Leboeuf-Yde C, 1996 [6]	Nordic countries	General population				✓	✓			
Morris LD, 2018 [7]	Africa	General population	✓			✓	✓			
Negash NA, 2022 [8]	Ethiopia	Healthcare professionals	✓	✓		✓		✓		✓
Park HJ, 2023 [9]	South Korea	General population							✓	
Sang S, 2021 [10]	Worldwide	Intensive care nurses				✓				
Shetty GM, 2022 [11]	India	Meta-analysis	✓			✓	✓			
Shmagel A, 2016 [12]	US	General population							✓	
Tesfaye AH, 2023 [13]	Africa	School teachers				✓				
Trompeter K, 2017 [14]	Worldwide	Athletes	✓				✓			
Walker BF, 2000 [15]	Worldwide	General population	✓			✓	✓			
Xing WY, 2024 [16]	Worldwide	Military personnel				✓				

Abbreviation: Ref = reference.

References

1. Almansour AH, Almutairi DS, Alaskar TM, et al. Prevalence of low back pain and disability among secondary school teacher in the eastern province of the Kingdom of Saudi Arabia: a cross-sectional analytical study. Front Public Health. 2024;12:1307845.
2. Chen C, Xiao B, He X, Wu J, Li W, Yan M. Prevalence of low back pain in professional drivers: a meta-analysis. Public Health. 2024;231:23–30.

3. Farahbakhsh F, Rostami M, Noormohammadpour P, et al. Prevalence of low back pain among athletes: A systematic review. J Back Musculoskelet Rehabil. 2018;31:901–16.
4. Hoy D, Bain C, Williams G, et al. A systematic review of the global prevalence of low back pain. Arthritis Rheum. 2012;64:2028–37.
5. Jia N, Zhang M, Zhang H, et al. Prevalence and risk factors analysis for low back pain among occupational groups in key industries of China. BMC Public Health. 2022;22:1493.
6. Leboeuf-Yde C, Klougart N, Lauritzen T. How common is low back pain in the Nordic population? Data from a recent study on a middle-aged general Danish population and four surveys previously conducted in the Nordic countries. Spine (Phila Pa 1976). 1996;21:1518–25.
7. Morris LD, Daniels KJ, Ganguli B, Louw QA. An update on the prevalence of low back pain in Africa: a systematic review and meta-analyses. BMC Musculoskelet Disord. 2018;19:196.
8. Negash NA, Tadele A, Jember Ferede A. Prevalence and Associated Factors of Low Back Pain Among Healthcare Professionals at University of Gondar Comprehensive and Specialized Hospital, Northwest Ethiopia: Cross-Sectional Study. J Pain Res. 2022;15:1543-52.
9. Park HJ, Choi JY, Lee WM, Park SM. Prevalence of chronic low back pain and its associated factors in the general population of South Korea: a cross-sectional study using the National Health and Nutrition Examination Surveys. J Orthop Surg Res. 2023;18:29.
10. Sang S, Wang J, Jin J. Prevalence of low back pain among intensive care nurses: A meta-analysis. Nurs Crit Care. 2021;26:476–84.
11. Shetty GM, Jain S, Thakur H, Khanna K. Prevalence of low back pain in India: A systematic review and meta-analysis. Work. 2022;73:429–52.
12. Shmagel A, Foley R, Ibrahim H. Epidemiology of Chronic Low Back Pain in US Adults: Data From the 2009-2010 National Health and Nutrition Examination Survey. Arthritis Care Res (Hoboken). 2016;68:1688–94.
13. Tesfaye AH, Abere G, Mekonnen TH, Jara AG, Aragaw FM. A systematic review and meta-analysis of low back pain and its associated factors among school teachers in Africa. BMC Musculoskelet Disord. 2023;24:499.
14. Trompeter K, Fett D, Platen P. Prevalence of Back Pain in Sports: A Systematic Review of the Literature. Sports Med. 2017;47:1183–207.
15. Walker BF. The prevalence of low back pain: a systematic review of the literature from 1966 to 1998. J Spinal Disord. 2000;13:205–17.
16. Xing WY, Zhang YH, Yang QH, Wang XQ. Prevalence and risk factors of low back pain in military personnel: a systematic review. EFORT Open Rev. 2024;9:1002–12.

S3 Appendix: Details regarding the selection process for indicators for the Brief Low Back Pain Questionnaire (B-LBPQ).

Regarding indicators for assessing the prevalence of LBP, many previous studies have reported the point, 1-year, and lifetime prevalence of LBP (**S2 Table**). Regarding indicators other than the prevalence of LBP, the Japan Low Back Pain Evaluation Questionnaire [1] and the Nordic Musculoskeletal Questionnaire [2] assessed the duration of LBP and its impact on daily activities. Furthermore, LBP is often classified based on symptom duration: Acute LBP is defined as lasting less than 4 weeks, subacute LBP as lasting 4 weeks to less than 3 months, and chronic LBP as lasting 3 months or longer [3]. Nationwide surveys in the United States [4] and South Korea [5] have investigated the prevalence of chronic LBP, defined as LBP persisting for at least 3 months.

Therefore, in this study, we adopted current pain, pain during the past 12 months, lifetime pain experience, pain duration, chronicity, and pain-related interference with daily activities as indicators of the B-LBPQ. Moreover, by using several items, the following reclassification was conducted. First, using the answers to questions 1 and 2, we divided the participants into three groups: No LBP, acute/subacute LBP (duration of less than 3 months), and chronic LBP (duration of 3 months or more). Next, using the answers to questions 4 to 6, LBP during the past 12 months was classified into the following five groups: no past-year LBP, past-year LBP < 30 days without activity restriction, past-year LBP < 30 days with activity restriction, past-year LBP ≥ 30 days without activity restriction, and past-year LBP ≥ 30 days with activity restriction.

To minimize respondent burden, those who reported no current LBP were exempt from answering questions 2 and 3. Those who reported that they had not experienced past-year LBP were exempt from answering questions 5 and 6.

References

1. Shirado O, Doi T, Akai M, Fujino K, Hoshino Y, Iwaya T. An outcome measure for Japanese people with chronic low back pain: an introduction and validation study of Japan Low Back Pain Evaluation Questionnaire. Spine (Phila Pa 1976). 2007;32:3052–9.
2. Kuorinka I, Jonsson B, Kilbom A, et al. Standardized Nordic questionnaires for the analysis of musculoskeletal symptoms. Appl Ergon. 1987;18:233–7.
3. Qaseem A, Wilt TJ, McLean RM, et al. Noninvasive Treatments for Acute, Subacute, and Chronic Low Back Pain: A Clinical Practice Guideline From the American College of Physicians. Ann Intern Med. 2017;166:514–30.
4. Shmagel A, Foley R, Ibrahim H. Epidemiology of Chronic Low Back Pain in US Adults: Data From the 2009-2010 National Health and Nutrition Examination Survey. Arthritis Care Res (Hoboken). 2016;68:1688–94.
5. Park HJ, Choi JY, Lee WM, Park SM. Prevalence of chronic low back pain and its associated factors in the general population of South Korea: a cross-sectional study using the National Health and Nutrition Examination Surveys. J Orthop Surg Res. 2023;18:29.

S4 Appendix: The translated version of the Brief Low Back Pain Questionnaire.

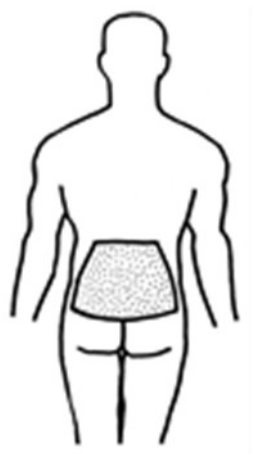

Explanation of Low Back Pain (LBP):

In this questionnaire, “low back pain” refers to pain or discomfort, such as aching or sharp pain, located anywhere within the area indicated in the figure on the left. This area extends from the lower margin of the ribs to the crease beneath the buttocks. Please include any pain that radiates or spreads into the leg(s) when answering the following questions.

	Questions	Response format
Q1	Do you have LBP today?<br>If “No” is selected, then skip to Question 4.	□ Yes<br>□ No
Q2	How long have you had your current episode of LBP?	□ < 4 weeks<br>□ 4 weeks to < 3 months<br>□ ≥ 3 months
Q3	How much does your current LBP make it difficult for you to do your daily activities (e.g., work, school, regular tasks, household chores, etc.)?	□ Not difficult<br>□ Mildly difficult<br>□ Moderately difficult<br>□ Considerably difficult<br>□ Extremely difficult
Q4	Have you had LBP at any time during the past 12 months?<br>If “No” is selected, then skip to Question 7.	□ Yes<br>□ No
Q5	What is the total length of time that you have had LBP during the past 12 months?	□ < 8 days<br>□ 8 to < 30 days<br>□ ≥ 30 days, but not every day<br>□ Every day
Q6	What is the total length of time that LBP has prevented you from doing your daily activities during the past 12 months?	□ 0 days<br>□ 1–7 days<br>□ 8 to < 30 days<br>□ ≥ 30 days
Q7	Have you ever had LBP?	□ Yes<br>□ No

Note: The original questionnaire was prepared and administered in Japanese. This English version is provided for reporting purposes and has not undergone formal cross-cultural validation.

S5 Fig: Distribution of RDQ scores among study participants.

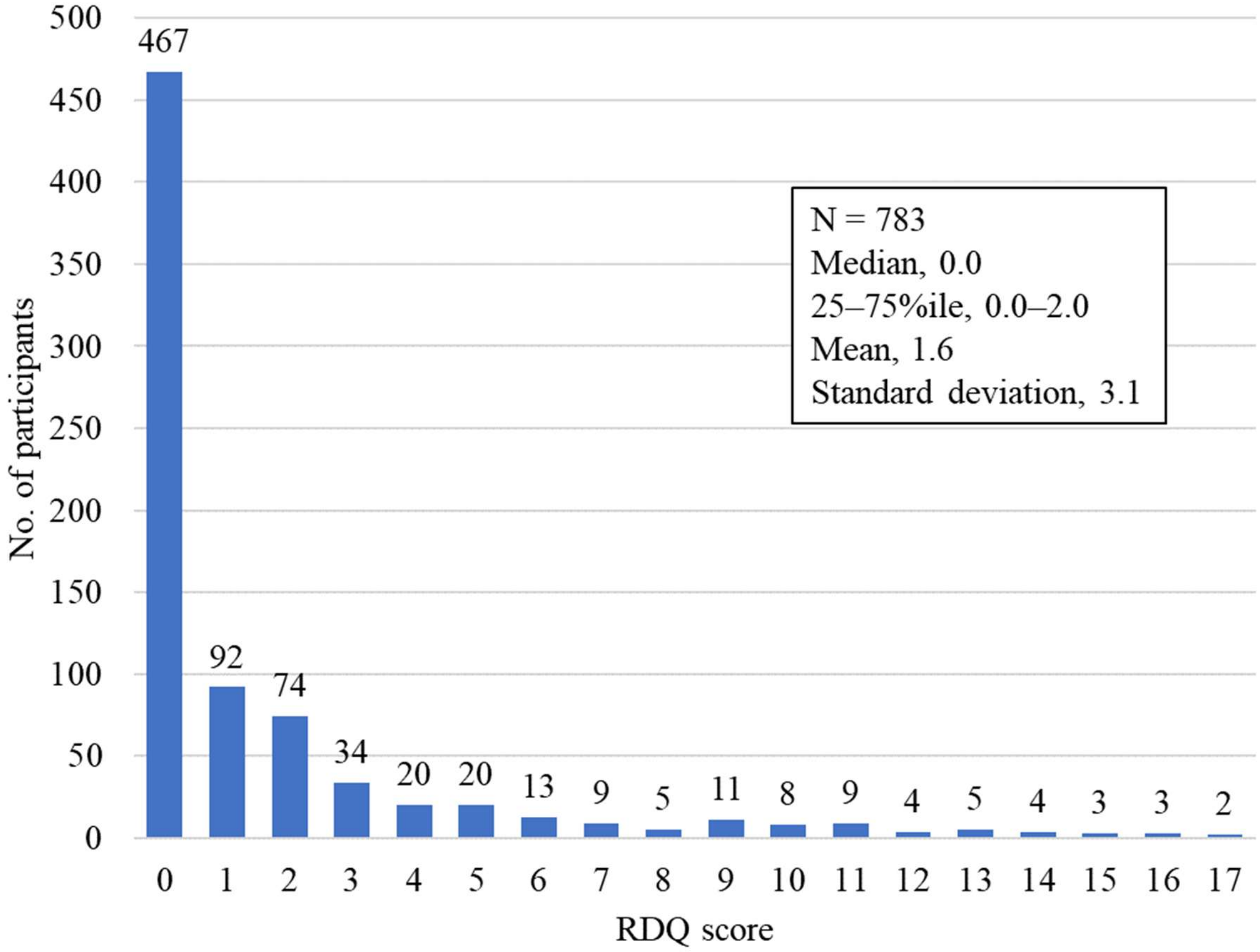

Abbreviation: RDQ = Roland–Morris Disability Questionnaire.

S6 Appendix: Distribution of responses in the test and retest: Questions 1–7.

Q1 Presence or absence of current LBP

1 Absent
2 Present

Test \ Retest	1	2	Total
1	319	53	372
2	36	187	223
Total	355	240	595

Q2 Duration of current LBP

1 <4 weeks
2 4 weeks to < 3 months
3 3 months or longer

Test \ Retest	1	2	3	Total
1	55	8	7	70
2	2	7	12	21
3	9	7	75	91
Total	66	22	94	182

Q3 Problems with daily activities due to current LBP

1 Not difficult
2 Mildly difficult
3 Moderately difficult
4 Considerably/extremely

Test \ Retest	1	2	3	4	Total
1	98	17	1	1	117
2	9	41	5	1	56
3	1	1	3	0	5
4	0	2	1	2	5
Total	108	61	10	4	183

Q4 Presence or absence of past-year LBP

1 Absent
2 Present

Test \ Retest	1	2	Total
1	104	38	142
2	17	427	444
Total	121	465	586

S6 Appendix: Continued.

Q5 Duration of past-year LBP

1 < 8 days
2 8 to < 30 days
3 30 days or longer, but not every day
4 Every day

Test \ Retest	1	2	3	4	Total
1	120	18	8	0	146
2	22	72	16	2	112
3	7	14	87	11	119
4	4	1	11	34	50
Total	153	105	122	47	427

Q6 Duration of disruption to daily activities due to past-year LBP

1 0 days
2 1–7 days
3 8 to < 30 days
4 30 days or longer

Test \ Retest	1	2	3	4	Total
1	185	33	4	1	223
2	31	90	10	7	138
3	1	6	27	5	39
4	2	3	5	14	24
Total	219	132	46	27	424

Q7 Lifetime experience of LBP

1 Absent
2 Present

Test \ Retest	1	2	Total
1	78	30	108
2	15	471	486
Total	93	501	594

Note: Those with missing data on each question were excluded from the analysis of each question. Questions 2 and 3 were limited to those with current LBP. Questions 5 and 6 were limited to those with past-year LBP.

Abbreviation: LBP = low back pain.

S7 Appendix: Distribution of responses in the test and retest: reclassification.

LBP assessment using questions 1 & 2

Presence or absence of chronic LBP

1 No chronic LBP
2 Chronic LBP

Test \ Retest	1	2	Total
1	458	29	487
2	24	75	99
Total	482	104	586

Chronic LBP assessment using questions 1 and 2

1 No LBP
2 Acute/subacute LBP
3 Chronic LBP

Test \ Retest	1	2	3	Total
1	319	40	10	369
2	27	72	19	118
3	8	16	75	99
Total	354	128	104	586

Past-year LBP assessment using questions 4 to 6

1 No past-year LBP
2 Past-year LBP < 30 days without activity restriction
3 Past-year LBP < 30 days with activity restriction
4 Past-year LBP 30 days or longer without activity restriction
5 Past-year LBP 30 days or longer with activity restriction

Test \ Retest	1	2	3	4	5	Total
1	104	27	9	1	0	141
2	14	115	25	7	4	165
3	0	25	66	0	15	106
4	1	8	1	55	8	73
5	1	3	13	6	72	95
Total	120	178	114	69	99	580

Note: Those with missing data on each question were excluded from the analysis of each question.
Abbreviation: LBP = low back pain.
