# Supplementary material for: Proposal and Initial Validation of a Brief Questionnaire for Epidemiological Assessment of Low Back Pain
Source: Pain Res Manag. 2026 Aug 25;2026:6203306. doi: 10.1155/prm/6203306 (PMC13504453; doi:10.1155/prm/6203306)
Supplement: Supplementary file 2 [file PRM-2026-6203306-s001.docx]

**STROBE Statement—Checklist of items that should be included in reports of cross-sectional studies**

Manuscript: Proposal and Initial Validation of a Brief Questionnaire for Epidemiological Assessment of Low Back Pain

Article type: Cross-sectional validation study with a two-week test-retest reliability component. Completed for resubmission to Pain Research and Management.

| **Section / Topic** | **Item No.** | **Recommendation** | **Reported on page / line(s)** | **Location / Comments** |
| --- | --- | --- | --- | --- |
| Title and abstract | 1a | Indicate the study design with a commonly used term in the title or the abstract. | Title, page 1, lines 1-2; Abstract Methods, page 2, lines 27-31. | The title describes the epidemiological assessment study, and the abstract states that this was a cross-sectional validation study. |
| Title and abstract | 1b | Provide in the abstract an informative and balanced summary of what was done and what was found. | Abstract, pages 2-3, lines 17-44. | Abstract includes Background, Methods, Results, and Conclusions, including the need for further validation. |
| Introduction: Background/rationale | 2 | Explain the scientific background and rationale for the investigation being reported. | Introduction, pages 4-5, lines 49-73. | Background explains the burden of low back pain, heterogeneity of definitions, and need for a brief questionnaire. |
| Introduction: Objectives | 3 | State specific objectives, including any prespecified hypotheses. | Introduction, page 5, lines 74-82; Methods, page 13, lines 204-214. | Objective is to propose and initially validate the B-LBPQ; construct validity hypotheses are described in Methods. |
| Methods: Study design | 4 | Present key elements of study design early in the paper. | Methods, page 6, lines 83-95. | The manuscript states the cross-sectional design for validity and the follow-up survey for test-retest reliability. |
| Methods: Setting | 5 | Describe the setting, locations, and relevant dates, including periods of recruitment, exposure, follow-up, and data collection. | Methods, pages 6-7, lines 87-104. | Data came from routine health checkups in October 2024 among employees in Osaka Prefecture; the follow-up survey occurred two weeks later. |
| Methods: Participants | 6a | Give the eligibility criteria, and the sources and methods of selection of participants. | Methods, pages 6-7, lines 96-104. | Participants were employees of a social welfare corporation with scheduled working hours of 30 hours or more per week. |
| Methods: Participants | 6b | For matched studies, give matching criteria and number of exposed and unexposed. | Not applicable. | This was not a matched study. |
| Methods: Variables | 7 | Clearly define all outcomes, exposures, predictors, potential confounders, and effect modifiers. Give diagnostic criteria, if applicable. | Methods, pages 7-12, lines 105-203; Table 1. | B-LBPQ items, chronic LBP definition, RDQ, covariates, physical workload, healthcare utilization, sleep restfulness, and K6 categories are described. |
| Methods: Data sources/measurement | 8 | For each variable of interest, give sources of data and details of methods of assessment. Describe comparability of assessment methods if more than one group. | Methods, pages 6-12, lines 87-203; Tables 1-2; S4 Appendix. | Data sources include routine health checkups and self-administered questionnaires; questionnaire translation and measurement details are described. |
| Methods: Bias | 9 | Describe any efforts to address potential sources of bias. | Methods, pages 6-7, lines 87-104; Discussion, page 25, lines 423-433; page 29, lines 478-481. | Opt-out procedure, comparison of test-retest participants with excluded participants, and limitations regarding selection bias and loss to follow-up are described. |
| Methods: Study size | 10 | Explain how the study size was arrived at. | Methods, pages 6-7, lines 96-104. | Potential participants were 784 eligible employees; 783 were included in construct validity analyses and 595 in test-retest reliability analyses. No formal sample-size calculation is reported. |
| Methods: Quantitative variables | 11 | Explain how quantitative variables were handled in the analyses. If applicable, describe which groupings were chosen and why. | Methods, pages 11-12, lines 171-203; Methods, pages 13-15, lines 204-249; Tables 1-2. | Age, K6, lifestyle variables, heavy handling, and B-LBPQ response categories are defined; regression adjustment variables and categorical analyses are described. |
| Methods: Statistical methods | 12a | Describe all statistical methods, including those used to control for confounding. | Methods, pages 13-15, lines 204-249; Table 5 note. | Non-parametric tests, kappa statistics, weighted kappa, PABAK, and multivariable modified Poisson regression with robust standard errors are described. |
| Methods: Statistical methods | 12b | Describe any methods used to examine subgroups and interactions. | Not applicable. | No subgroup or interaction analyses are reported. |
| Methods: Statistical methods | 12c | Explain how missing data were addressed. | Methods, page 15, lines 250-264; Table 2 note; Table 5 note. | Missing covariate values were imputed using multiple imputation by chained equations; missing B-LBPQ responses were handled by complete-case analysis. |
| Methods: Statistical methods | 12d | If applicable, describe analytical methods taking account of sampling strategy. | Not applicable. | No complex sampling strategy is reported. |
| Methods: Statistical methods | 12e | Describe any sensitivity analyses. | Not applicable. | No sensitivity analyses are reported. |
| Results: Participants | 13a | Report numbers of individuals at each stage of study. | Methods, pages 6-7, lines 96-104; Results, page 17, lines 278-287; S1 Table. | The manuscript reports 784 potential participants, 783 in construct validity analyses, and 595 in test-retest analyses. |
| Results: Participants | 13b | Give reasons for non-participation at each stage. | Methods, pages 6-7, lines 87-104; S1 Table. | The manuscript reports the opt-out/informed-consent procedures and compares included and excluded participants for test-retest reliability; detailed reasons for non-participation are not reported. |
| Results: Participants | 13c | Consider use of a flow diagram. | Not provided. | A flow diagram is not included; the participant numbers are reported in the text and S1 Table. |
| Results: Descriptive data | 14a | Give characteristics of study participants and information on exposures and potential confounders. | Results, page 17, lines 278-287; Table 2. | Demographic characteristics and covariate distributions are reported. |
| Results: Descriptive data | 14b | Indicate number of participants with missing data for each variable of interest. | Methods, page 15, lines 250-264; Table 2; Tables 3-5 notes. | Missing values are reported in Table 2 and analysis-specific missing values are reported in the tables. |
| Results: Descriptive data | 14c | Summarise follow-up time, if applicable. | Methods, pages 6-7, lines 89-104; Table 4. | The test-retest interval was two weeks; otherwise this was a cross-sectional study. |
| Results: Outcome data | 15 | Report numbers of outcome events or summary measures. | Results, pages 17-19, lines 278-324; Tables 3-5. | Prevalence of current, past-year, lifetime, and chronic LBP, RDQ distributions, and reliability measures are reported. |
| Results: Main results | 16a | Give unadjusted estimates and, if applicable, confounder-adjusted estimates and their precision. Make clear which confounders were adjusted for and why. | Results, pages 17-19, lines 288-324; Table 3; Table 5 note. | Median RDQ scores and adjusted prevalence ratios with 95% CIs are reported; adjustment variables are stated in Table 5 note and Methods. |
| Results: Main results | 16b | Report category boundaries when continuous variables were categorized. | Methods, pages 11-12, lines 198-203; Tables 1-2; Table 5. | K6, age, heavy handling, treatment frequency, sleep restfulness, and B-LBPQ categories are reported. |
| Results: Main results | 16c | If relevant, consider translating estimates of relative risk into absolute risk for a meaningful time period. | Not applicable. | The study reports prevalence ratios for cross-sectional outcomes; no meaningful time period for absolute risk translation is applicable. |
| Results: Other analyses | 17 | Report other analyses done, such as analyses of subgroups and interactions, and sensitivity analyses. | Results, pages 17-19, lines 288-324; Tables 3-5; S1 Table. | Construct validity, test-retest reliability, internal consistency, known-groups validity, and comparison of included/excluded test-retest participants are reported. |
| Discussion: Key results | 18 | Summarise key results with reference to study objectives. | Discussion, pages 19-21, lines 325-353; pages 21-23, lines 354-386. | Key validity, reliability, and measurement findings are summarized in relation to the B-LBPQ objective. |
| Discussion: Limitations | 19 | Discuss limitations, taking into account sources of potential bias or imprecision. Discuss both direction and magnitude of any potential bias. | Discussion, pages 24-29, lines 406-487. | Limitations include two-week interval, selection bias, generalizability, lack of cross-cultural validation, no pain intensity, limited psychometric properties, temporal constructs, loss to follow-up, and single-item activity interference. |
| Discussion: Interpretation | 20 | Give a cautious overall interpretation of results considering objectives, limitations, multiplicity of analyses, results from similar studies, and other relevant evidence. | Discussion, pages 19-29, lines 325-493; Conclusions, pages 29-30, lines 495-501. | Interpretation is cautious and frames the B-LBPQ as an initial/preliminary tool requiring further validation. |
| Discussion: Generalisability | 21 | Discuss the generalisability (external validity) of the study results. | Discussion, pages 24-29, lines 406-493; Conclusions, pages 29-30, lines 495-501. | The manuscript discusses limited generalizability to other occupational groups, age groups, general populations, and non-Japanese settings. |
| Other information: Funding | 22 | Give the source of funding and the role of the funders for the present study and, if applicable, for the original study on which the present article is based. | Funding statement, page 31, lines 526-527. | Funding source is reported as the Center Research Expense of Nara Medical University; no grant number is stated. |

Note: This checklist was completed using the revised manuscript files prepared for major revision. Page and line numbers refer to the revised main manuscript unless otherwise stated. Items marked not applicable are included to preserve the standard STROBE cross-sectional checklist structure.

Source checklist: STROBE Statement checklist for cross-sectional studies.
